# Indexing a protein-protein interaction network expedites network alignment

**DOI:** 10.1186/s12859-015-0756-0

**Published:** 2015-10-09

**Authors:** Md Mahmudul Hasan, Tamer Kahveci

**Affiliations:** Department of Computer & Information Science and Engineering, University of Florida, Gainesville FL, 32611 USA

**Keywords:** Network alignment, Protein-protein interaction network, Indexing

## Abstract

**Background:**

Network query problem aligns a small query network with an arbitrarily large target network. The complexity of this problem grows exponentially with the number of nodes in the query network if confidence in the optimality of result is desired. Scaling this problem to large query and target networks remains to be a challenge.

**Results:**

In this article, we develop a novel index structure that dramatically reduces the cost of the network query problem. Our index structure maintains a small set of reference networks where each reference network is a small, carefully chosen subnetwork from the target network. Along with each reference, we also store all of its non-overlapping and statistically significant alignments with the target network. Given a query network, we first align the query with the reference networks. If the alignment with a reference network yields a sufficiently large score, we compute an upper-bound to the alignment score between the query and the target using the alignments of that reference and the target (which is stored in our index). If the upper-bound is large enough, we employ a second round of alignment between the query and the target by respecting the mapping found in the first alignment.

Our experiments on protein-protein interaction networks demonstrate that our index achieves a significant speed-up in running time over the state-of-the-art methods such as ColT. The alignment subnetworks obtained by our method are also statistically significant. Finally, we observe that our method finds biologically and statistically significant alignments across multiple species.

**Conclusions:**

We developed a reference network based indexing structure that accelerates network query and produces functionally and statistically significant results.

## Background

Biological networks describe how different molecules (such as proteins or gene products) interact with each other to carry out various cellular functions. Depending on the interacting molecules and their interaction types, biological networks are often classified into several categories such as gene regulatory networks, signaling networks or protein-protein interaction networks. One common way to model such networks is to represent them as graphs, where nodes and edges denote the molecules and interactions respectively.

Comparative analysis of biological networks is one of the most fundamental techniques in understanding how cells function [1]. The first step in such analysis is to identify the similarity between pairs of biological networks by aligning them. An alignment of two networks maps the nodes and edges of one network to those of the other. The similarity between two networks is often modeled as a function of the similarity between the aligned nodes, matching edges, and possible insertion or deletion of nodes. We formally define the concept of similarity later in this article. Network alignment has already been successfully used in many applications including identification of functional annotations [2], and reconstructing biological networks from newly sequenced genome [3], among many others.

We can categorize existing literature on network alignment into two classes: (i) alignment of the entire networks (known as *global* alignment) [4, 5], and (ii) alignment of smaller subnetworks among the networks being compared (known as *local* alignment) [6–8]. In the context of local alignment, when a small query network is matched with a large target network, the problem is known as the *network query* problem [9, 10]. In this article, we focus on the network query problem. The complexity of the network alignment problem stems from its close relationship with graph and subgraph isomorphism problems, which are GI-Complete [11] and NP-Complete [12] respectively. Thus, finding an accurate solution to this problem remains to be impractical as the size of the given networks grow. Existing solutions often follow one of the following two approaches: (i) *heuristic* solutions find a best-effort alignment network but provide no guarantee about the optimality of the result [4, 5, 13]; (ii) *approximate* solutions find an alignment subnetwork and provide provable confidence bound in the optimality of the result [6, 10]. Heuristic methods are often faster, whereas approximate methods often employ expensive dynamic programming methods to get the result. In this article, we focus only on approximate methods for network query problem as they provide provably accurate results.

The effectiveness of the approximate methods is limited by the number of nodes in the query and target networks, particularly with that of the query network. The complexity of these methods increase exponentially with the number of nodes in the query network. This bottleneck becomes even more catastrophic as the target networks are often as large as the entire biological networks and thus are very large in size. As a result, there is an urgent need for efficient yet accurate method for querying of large network databases. Indexing has been traditionally used for accelerating query processing in relational databases [14, 15]. It has also been applied to *similarity search* in biological network databases. We discuss these methods in Section ‘Related work’.

### Contributions

In this article, we develop a new and scalable method to solve the network query problem efficiently for large target networks. Our method is based on the observation that biological networks often contain conserved subnetworks also knows as *motifs* [16–18]. Following from that observation, at the heart of our method lies a novel reference-based index structure we develop here. Briefly, we start by selecting a *small* set of representative subnetworks, called *reference networks* or simply *references*, sampled from the target biological network. We choose these references in a way that ensures that they collectively summarize the topology of the target network. We store the non-overlapping, significant mappings of these reference networks in the target network as our index structure.

**Fig. 1:**
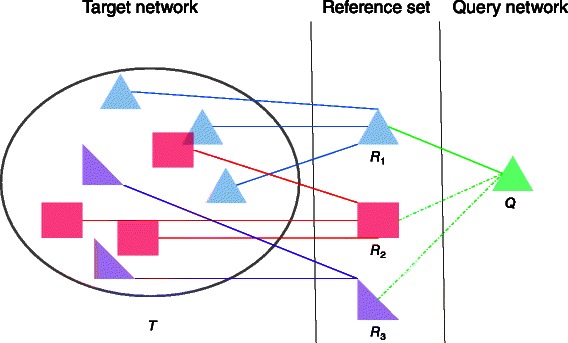
Overview of the reference-based indexing used in this article. We select three reference networks *R*_1_, *R*_2_, and *R*_3_ from the target network *T*, and {*R*_1_,*R*_2_,*R*_3_} is the reference set. Reference networks *R*_1_, *R*_2_, and *R*_3_ have three, three and two non-overlapping and significant mappings in *T* respectively. Given a query network *Q*, we align *Q* with all the references *R*_*i*_ respectively. Here, *Q* only aligns with *R*_1_ successfully. We use this alignment to find an alignment of *Q* with *T* using the three mappings of *R*_1_ in *T*

Figure 1 shows the overview of the proposed method on a toy example. In this figure, we select three reference networks, namely *R*_1_, *R*_2_, and *R*_3_ that summarize the target network *T*. Reference networks *R*_1_, *R*_2_, and *R*_3_ have three, three and two significant mappings in the target network respectively. Given a query network, we first align the query to the references one by one. If a reference network yields a statistically significant alignment with the query, we compute an upper bound to the alignment score between the query and the target network that can be found using this reference network. If this upper bound is large enough, we investigate the non-overlapping significant mappings of that reference. The index provides an *indirect* mapping from the query to the target via the reference. We then compute a local alignment that respects this indirect alignment to maximize the alignment score.

In Fig. 1, the query network *Q* aligns with the reference network *R*_1_. We can use the three non-overlapping, significant mappings of *R*_1_ to find an indirect mapping of *Q* with *T* using *R*_1_. Note that the proposed method requires a pairwise network alignment algorithm to map the query and reference networks. For this purpose, we use ColT [10] as it is currently the fastest method that finds alignment subnetworks with provable confidence in the optimality of the result. That said, our indexing scheme is independent of any specific underlying network query algorithm. Thus, one can replace ColT with any other alignment algorithm without altering the rest of our indexing method. Our experiments on real protein-protein interaction (PPI) networks demonstrate that the proposed method is over 20 times faster than ColT. Furthermore, our experiments show that the resulting alignments are statistically significant.

We organize the rest of this article as follows: Section ‘Related work’ discusses existing works in the literature. Section ‘Method’ describes our method. Section ‘Results and discussion’ presents the experiments and results. We conclude this article in Section ‘Conclusion’.

## Related work

Network alignment problem can be considered into two broad categories, namely local and global network alignment. Local network alignment finds similar subnetworks among the networks being compared based on some similarity criteria (i.e., defined by the topological similarity among the subgraphs and the homological similarity of the constituent nodes) [8, 9, 19]. The *network query* problem is a related problem in the context of local alignment [9, 20]. It matches a small query network to a large target network to find a subnetwork in the target that is similar to the query network. PathBLAST [7], TALE [21], ABINET [22, 23] fit in this category. PathBLAST queries a simple path against a target network and returns a ranked list of matching paths and the overlaps among them in the target. ABINET uses the well characterized PPI network as a fingerprint (called ‘Master’) and guides the alignment to the second network (called ‘Slave’). The resulting alignments also retain the structural characteristics of the Master. Note that all these methods find the matching subnetworks based on their similarity criteria; but do not provide any provable confidence in the optimality of the results. On the other hand, several existing alignment methods provide confidence in the optimality of the matching subnetwork by employing expensive dynamic programming. These methods perform the three basic network edit operations, namely (i) matching a query node to a node in the target network, (ii) inserting a node in the alignment, and (iii) deleting a node from the query network to maximize the alignment score of the corresponding alignment subnetwork. This limits the practicality of the alignment methods, since complexity of these methods usually depend on the topology and the size of the query network. More specifically, let us denote the number of nodes in the query and the target with *m* and *n* respectively. Even for simple query topologies such as simple paths and cycles, the complexity of this problem is *O*(*n* ^*m*^), where each potential sub-alignment should maintain the potential list of already visited nodes. These methods use a randomization technique called *color-coding* [24]. This allows one to maintain the list of visited colors at considerably lower complexity compared to the list of visited nodes (i.e., *O*(*n* ^*m*^)). It evaluates a subset of subnetworks as potential alignment subnetwork, and repeat the process multiple times to get the user specified confidence in the optimality of the alignment subnetwork. *Due to the complexity of the alignment algorithm, existing methods do not find the alignment for arbitrary query network topology.* For instance, QPath [25] finds the alignment of a linear pathway in a target network. QNet and ColT extend the topology of the query network to be of tree topology [6, 10]. These methods can act as building block to any indexing technique that provides provable confidence bound in the quality of the result.

In global network alignment problem, two networks are compared as a whole to find the best matching between the nodes of the networks in terms of some scoring function. IsoRank algorithms are among the earliest global network alignment methods [4, 26]. They estimate node similarity by recursively defining similarity among the neighbors of the nodes. GRAAL series of network alignment methods use *graphlet degree signatures* for topological similarity [5, 27, 28]. In summary, all the alignment methods (both local and global) use topological and homological information to score an alignment. However, these methods vary widely in the way they use these information.

Sometimes, network alignment is sought for a query network against a database of biological networks. We can find the best match of the query in each of the database networks. Alternatively, we can return only the database networks with which the similarity of the query network is over a user specified threshold. This problem is known as the *similarity search* problem. Note that the similarity search does not attempt to find the best alignment. It rather seeks if the similarity score is above a threshold for each database network.

Building an index over the database can accelerate similarity search. We can broadly classify the existing indexing techniques for this problem into three groups as *feature-based*, *tree-based*, and *reference based* index. Feature-based indexing methods extract specific features (e.g., path, small subgraphs etc.) from the database networks and index them. When a query is issued, they extract features in the query and match those to features in the index. Comparing features is a significantly cheaper task than network alignment. Thus, this strategy is preferable if it can filter a large number of database networks. Many indexing techniques fit in this category [21, 29–31]. TALE [21] matches large query networks against a database of target networks. To tackle large query networks, TALE introduces an index named the Neighborhood Index (NH-Index) based on the induced subgraph of a node and it’s neighbors. It computes the ‘important nodes’ in the query and probes the index to find its matches in the targets. It then *extends* the matching based on those ‘anchor points’. The notion of ‘important’ node stems from degree centrality and the matching between the nodes are based on the node/group labels. It ignores the sequence contents of the nodes while matching nodes. Additionally, the match-and-extend based matching tool does not provide confidence in the optimality of the result. SAGA [29], on the other hand, provides a flexible approximate graph matching tool for smaller query networks. It allows nodes to match to orthologous groups only. However, the corresponding matching algorithm is also inadequate to provide confidence bound in the optimality of the result. Closure-Tree(C-Tree) is a representative of tree-based indexing technique [32]. C-Tree stores the index structure as a tree. It places all the database networks at the leaf level of this tree. Each internal node is a network that is the union of all the networks at the leaf nodes of the subtree rooted at that node. When a query is issued, it aligns the query to the network stored at the root node of the index. If the alignment score is large enough, it explores the children nodes iteratively. Otherwise, it prunes the entire subtree rooted at that node. This strategy has no affect in reducing the cost of alignment in a single target. It is identical to the pairwise network alignment problem. Reference-based indexing is similar to feature-based indexing. It samples small, random subgraphs (knows as references) from the database networks and index them. When a query is issued, it aligns the query network with all the references. It computes an upper and a lower bound to the alignment score of each database network and filters those with a lower upper bound than the user specified threshold. RINQ [33] fits into this category. Functional summary of a network can also be used as index. FUSE [34] generates functional maps (clustering) of a PPI network at different levels of granularity. It presents the underlying PPI network as a *functional summary* graph of interconnected functional modules. Recent works such as DualAligner [35] incorporates functional annotation (e.g., Gene Ontology (GO) terms) informations with the interaction data to align networks in multiple granularities. DualAligner performs protein-to-protein alignments when detailed protein-protein alignment can be ascertained, and performs functional region-to-region alignment when data confidence is low. DualAligner also does not provide confidence in the optimality of their alignments.

Note that the similarity search problem is different than the network query problem considered in this article, and the above mentioned indexing techniques are not applicable in this context. Here, we propose a novel reference based indexing method that finds alignment in a target network with high confidence in the optimality of the result. We use ColT as the building block in our indexing technique, hence, we find alignment of the query networks of tree topology. Having said that, our indexing method is generic to be applied for any topology.

## Method

Network query problem seeks the optimal alignment of a query network in an arbitrarily large target network. This is a nontrivial task as the complexity of the underlying problem grows exponentially with the size of the query network (i.e., number of nodes). Here, we present a new method that addresses this challenge. The core idea behind our method is the novel index structure we develop to summarize the target network. We start by introducing the essential notation needed to describe our method in Section ‘Preliminary notation’. We present an overview of our method in Section ‘Overview of our method’. We discuss how we construct the index structure in Section ‘Index creation’. We explain how we utilize this index to align a given query to target network in Section ‘Query processing’.

### Preliminary notation

We denote the query network by *Q*=(*V*_*Q*_,*E*_*Q*_) and the large target network by *T*=(*V*_*T*_,*E*_*T*_). An alignment of *Q* with *T* maps the nodes of *Q* to those of a connected subnetwork of *T*. An alignment can lead to insertion of new nodes into *Q* or deletion of some of the existing nodes of *Q*. We model an alignment of *Q* with *T* using a bijection function *α*:*V*_*Q*_∪{*∅*}→*V*_*T*_∪{*∅*}, where *∅* represents insertion/deletion (*indel* for short) of nodes. We say that an interaction of *Q* denoted with the edge (*u*_*Q*_,*v*_*Q*_)∈*E*_*Q*_ is aligned with an interaction of *T* denoted with (*u*_*T*_,*v*_*T*_) if *α*(*u*_*Q*_)=*u*_*T*_ and *α*(*v*_*Q*_)=*v*_*T*_.

Following from the state-of-the-art methods in the literature [6, 10], we measure the quality of an alignment based on three factors: (i) similarity between the matching node pairs, (ii) weight of the aligned interactions, and (iii) penalty incurred due to insertion/deletion of nodes. More specifically, let us denote the similarity between the nodes *u*∈*V*_*Q*_ and *v*∈*V*_*T*_ with *s* *i* *m*(*u*,*v*), and weight of the aligned interaction (*u*,*v*)∈*E*_*T*_ with *w*(*u*,*v*) if the target network is weighted. Also, let us denote the penalty incurred for each node insertion and deletion with *δ*_*i*_ and *δ*_*d*_ respectively. Using this notation, we compute the score of an alignment *α*, that incurs *n*_*i*_ insertions and *n*_*d*_ deletions as:

$$\begin{aligned} \text{alignment-score}(\alpha) &= \sum\limits_{\substack{u \in V_{Q}\\ \alpha(u) \in V_{T}}}{sim(u, \alpha(u))} \\ &\quad+ \sum\limits_{\substack{(u, v) \in E_{Q}\\ (\alpha(u), \alpha(v)) \in E_{T}}}{w(\alpha(u), \alpha(v))} \\ &\quad+n_{i} \delta_{i} + n_{d} \delta_{d} \end{aligned} $$

The optimal alignment of *Q* and *T* is the mapping that results in the highest alignment score. Let us denote the optimal alignment by *α* ^∗^ (i.e., *α* ^∗^= arg max*α*{alignment-score(*α*)}). The alignment *α* ^∗^ defines a subnetwork $T^{\prime } = (V_{T^{\prime }}, E_{T^{\prime }})$ of *T*, where $V_{T^{\prime }} =\{v|~v \in V_{T}$ and ∃*u*∈*V*_*Q*_ such that *α* ^∗^(*u*)=*v*} and $E_{T^{\prime }}=$ { (*u*,*v*)| *u*,*v*∈*V*_*T*_ and (*u*,*v*)∈*E*_*T*_}. We call *T* ^′^ the *alignment subnetwork*.

### Overview of our method

In the literature, numerous index structures have been developed to accelerate querying relational databases [14, 15]. However, this strategy has almost never been utilized for querying biological networks. This is mainly due to the fact that the complexity of the network topologies, which is not observed in relational databases, makes it an extremely hard problem. This article introduces a new angle to the network querying problem by building an index structure over the target network *T*. With the help of this index structure we dramatically reduce the computational cost of the network query problem. In order to describe our method, we first need to answer two key questions:

(i) How do we build the index structure?
(ii) How do we use the index structure to align a given query with the target network?

We summarize the answers to these questions next. We elaborate on these answers in Sections ‘Index creation’ and ‘Query processing’ respectively. Table 1 lists the notation we use in the rest of this article.

**Table 1:** Symbols used in this article

Symbol	Meaning
*Q*	Query network
*T*	Target network
$\mathcal {I}$	Initial reference network set
$\mathcal {F}$	Final reference network set
*R*_*i*_	*i*th reference network
*ϕ*_*ij*_	*j*th mapping of the reference *R*_*i*_ with *T*
*Φ*_*i*_	{*ϕ*_*ij*_}, i.e.,set of the mappings of reference *R*_*i*_ with *T*
*Φ*	{*Φ*_*i*_}, i.e., set of all mappings

**Index construction** We use a set of small networks, called *reference networks* (or simply *references*), to index the given target network *T*. We choose reference networks through repetitive application of random walks on *T*. Let us denote the set of *n* such references obtained at the end of *n* random walks with $\mathcal {I} = \{R_{1}, R_{2}, \ldots, R_{n}\}$. We call $\mathcal {I}$ the *initial reference set*. For each reference $R_{i} \in \mathcal {I}$, we compute its alignment subnetwork $T^{\prime } = (V_{T^{\prime }}, E_{T^{\prime }})$ in *T*. We then update *T* by removing $V_{T^{\prime }}$ from *V*_*T*_ and find the next alignment subnetwork of *R*_*i*_ in *T*. We repeat this procedure to find alternative alignments of *R*_*i*_ in *T* as long as the score of the alignment subnetwork is *statistically significant*. We defer the formal description of statistical significance to Section ‘Results and discussion’. This strategy ensures that the generated alignment subnetworks are non-overlapping and statistically significant. The union of the set of all the nodes in the alignment subnetworks of *R*_*i*_ is the *coverage* of *R*_*i*_ on *T*, and we denote it by *c* *o* *v* *e* *r*(*R*_*i*_). Notice that although the alignment subnetworks of a given reference *R*_*i*_ are guaranteed to be mutually exclusive, those of different references *R*_*i*_ and *R*_*j*_ may overlap with each other. As we explain later in detail in Section ‘Index creation’, this overlap creates redundancy in the index. To avoid redundancy, we select a smaller subset of references from this set so that they maximize the total coverage of *T*. We denote the resulting final reference set by $\mathcal {F}$ ($\mathcal {F} \subseteq \mathcal {I}$).

**Querying** Once the index is created for the target network *T*, we are ready to query that network using the index. Given a query network *Q*, we first align *Q* with each reference network $R_{i} \in \mathcal {F}$. This alignment is computationally inexpensive since the number of nodes in both query and reference networks are small. The alignment between *Q* and *R*_*i*_ leads to a mapping *ψ* (i.e., $(\psi : V_{Q} \cup \{\emptyset \} \rightarrow V_{R_{i}} \cup \{\emptyset \})$) between the nodes of *Q* and *R*_*i*_ with possible indels. Recall that for each reference network *R*_*i*_, our index stores its non-overlapping alignments with the target network. Assume that such a mapping is denoted by *ϕ* where $(\phi : V_{R_{i}} \cup \{\emptyset \} \rightarrow V_{T} \cup \{\emptyset \})$. Using *ϕ*, we quickly find a mapping between the query and the target network as *ϕ*(*ψ*()) (details in Section ‘Query processing’). We then compute an upper-bound to the alignment score between *Q* and *T* using the resulting mapping. If the upper-bound score is smaller than the current best alignment score, we ignore this mapping. Otherwise, we further optimize this mapping by carefully realigning the nodes of *Q* based on the current mapping *ϕ*(*ψ*()). We iterate over all the reference networks and their alignment subnetworks in the index and report the best result among them.

### Index creation

The set of reference networks ideally should possess two desirable characteristics: (i) They should summarize the target network well, and (ii) aligning a query network with those in the reference set should be computationally inexpensive. Here, the former characteristic aims at maximizing accuracy of the result while minimizing the cost of query and target network alignment. The latter aims to minimize the additional alignment cost introduced by the references. We create such a reference set in two steps: In the first step, we generate an initial set of reference networks, denoted with $\mathcal {I}$, which ensures that the first characteristic holds while disregarding the second one. In the next step, we filter a subset of the reference networks from the initial set to ensure that the second characteristic is also satisfied without violating the first one. We call the resulting set of references the *final reference network set*, and denote it with $\mathcal {F}$. Algorithm 1 presents a pseudo-code of these two steps. We elaborate on these steps next.



**Step I. Construction of initial reference set. (lines 2-6 of Algorithm 1)**

Let *k* be a user supplied positive integer denoting the number of nodes in each reference network. We create each reference network $R \in \mathcal {I}$ by performing a random walk on the the target network *T* as follows: We start by randomly selecting a node in *T*. This node is the seed of the reference *R*. We then grow *R* by randomly inserting one of the incident edges in *T* to a node in *R* along with the node connected to that edge. We repeat this process until *R* has *k* nodes.

Note that the choice of the value of the parameter *k* is governed by several factors. First, the network alignment problem considered in this article is prohibitively expensive. More specifically, the time and space complexity rises exponentially with the number of nodes in the query [6, 10]. For large confidence values (such as 90 % or more), solving this problem becomes impractical beyond query networks of eight or nine nodes [10]. This imposes a practical limit on the size of the reference networks. Second, we expect this value to be “close” (not necessarily identical) to the size of the query networks. Thus the underlying user needs also govern the value of *k*. Therefore, we want the reference networks to represent the future query networks well and want the alignment with the query networks to be fast. If *k* is large, it has the possibility of finding more indirect matching with the query; but the alignment will be expensive. On the other hand, if *k* is much smaller, the alignment will be fast; but the quality of the indirect matching will suffer. These two points set a range of values for the number of nodes *k* a reference network should have. As long as the reference network promises a good alignment and the size is manageable, the reference is considered good.

Once a reference network $\phantom {\dot {i}\!}R_{i} = (V_{R_{i}}, E_{R_{i}})$ is constructed, we compute its non-overlapping, significant alignment subnetworks in *T* iteratively. Let us denote the *j*th non-overlapping alignment between *R*_*i*_ and *T* with the function *ϕ*_*ij*_():*R*_*i*_→*T* (i.e., *ϕ*_*ij*_(*u*)=*v* means that node *u* of *R*_*i*_ is aligned with node *v* of *T*). Note that the mapping *ϕ*_*ij*_() may introduce indels in the alignment. For example, when a node *u* in *R*_*i*_ deleted in an alignment, *ϕ*_*ij*_(*u*) will be set to *∅*.

Now, we are ready to describe how we construct the alignments *ϕ*_*ij*_() of the reference *R*_*i*_. We use the ColT algorithm [10] to align *R*_*i*_ with *T* optimally with a high confidence (we set confidence parameter to 99 %). We prefer this algorithm as it is currently the fastest method that ensures the optimality of the result with a provable confidence. It is worth noting that one can replace ColT with another algorithm without changing the rest of our method. If the resulting alignment between *R*_*i*_ and *T* is statistically significant (see Section ‘Results and discussion’ for definition of statistical significance), we store the resulting mapping *ϕ*_*i*1_ as the first mapping function of *R*_*i*_ with *T*. We then remove the nodes in the alignment subnetwork of *ϕ*_*i*1_ from *T* along with the edges incident to them. We iteratively align *R*_*i*_ with the reduced network *T* to find the next best alignment *ϕ*_*i*2_. We repeat this process of obtaining new alignments and reducing *T* until no new statistically significant alignment can be found between *R*_*i*_ and *T*. Let us denote the number of such alignments of *R*_*i*_ with *π*_*i*_. We denote the set of non-overlapping significant alignments with $\phantom {\dot {i}\!}\Phi _{i} = \{\phi _{i1}, \phi _{i2}, \ldots, \phi _{i\pi _{i}}\}$.

Once the first reference *R*_1_ along with its set of alignments *Φ*_1_ is constructed, we repeat the same procedure to construct more references *R*_2_, *R*_3_, … along with their sets of alignments *Φ*_2_, *Φ*_3_, … until the *stopping criterion* is met. In order to describe the stopping criteria, we first introduce the concept of *coverage*. Consider the initial reference set $\mathcal {I} = \{R_{1}, R_{2}, \ldots, R_{n}\}$. Briefly, the coverage of $\mathcal {I}$ is the set of nodes of *T* that map to at least one node of at least one reference in $\mathcal {I}$ through their statistically significant alignments. Mathematically, we denote the coverage of *R*_*i*_ as $\phantom {\dot {i}\!}cover(R_{i}) = \{v| \exists \phi _{\textit {ij}} \in \Phi _{i}~\text {and}~\exists u \in V_{R_{i}}~\text {such that}~\phi _{\textit {ij}}(u) = v\}$. Similarly, we denote the coverage of $\mathcal {I}$ as $cover(\mathcal {I}) = \cup _{R_{i} \in \mathcal {I}}cover(R_{i})$. We stop creating new reference networks as soon as $|cover(\mathcal {I})|$ reaches a user supplied *η* % of the total number of nodes in *T*. For instance, if *T* has 100 nodes and *η*= 60 %, we stop inserting new reference network in $\mathcal {I}$ as soon as $cover(\mathcal {I})$ reaches to at least 60 nodes in the target network.

**Step II. Construction of the final reference set. (lines 7-12 of Algorithm 1)**

We conjecture that as the number of nodes in $cover(\mathcal {I})$ increases, chances that it will lead to the optimal alignment of a given query increases. We defer discussion of the rationale behind this conjecture to Section ‘Query processing’. Clearly, as the number of references in set $\mathcal {I}$ grows, coverage of $\mathcal {I}$ monotonically grows as well. On the other hand, each reference in $\mathcal {I}$ increases the cost of aligning query with the target network as we first align query with the references (see Section ‘Query processing’). Thus, it is desirable to have a small reference set with a large coverage. In this step, we resolve this conflict between accuracy and the running time performance. More specifically, we would like to choose a subset $\mathcal {F}$ of the candidate reference set $\mathcal {I}$, such that the coverage of $\mathcal {F}$ on *T* is at least a given coverage cutoff and the size of $\mathcal {F}$, (i.e., $|\mathcal {F}|$), is as small as possible. This is the classic set cover problem which is NP-Complete [12]. We use a greedy technique to solve this problem efficiently. For each reference network $R_{i} \in \mathcal {I}$, we construct the set *c* *o* *v* *e* *r*(*R*_*i*_). We iteratively create the final reference set $\mathcal {F}$ that yields the desired coverage as follows: We start by initializing the final reference set $\mathcal {F}$ and $cover(\mathcal {F})$ as the empty set. At each iteration, we select the reference network $R_{i} \in \mathcal {I}$ such that the value of $cover(\mathcal {F} \cup \{R_{i}\})$ is the largest. We then remove *R*_*i*_ from $\mathcal {I}$, insert it to $\mathcal {F}$ and update $cover({\mathcal {F}})$. We repeat this iterative process to move the next reference from $\mathcal {I}$ into $\mathcal {F}$ until $|cover(\mathcal {F})|$ reaches to at least *η*% of the target network size. This solution to the set cover problem results in a $O(\ln (|\mathcal {I}|))$ approximation to the optimal solution of the problem which is the best for a polynomial solution unless *P*= NP [36].

### Query processing

Once the final reference network set $\mathcal {F}$ is generated along with the set of mappings *Φ*, we use it to align *Q* with *T*. Briefly, we first align *Q* with each reference *R*_*i*_ in $\mathcal {F}$. We then quickly get an initial *indirect* alignment of *Q* with *T* with the help of each *Φ*_*i*_∈*Φ*. Next, we compute an upper bound to the score of the best alignment we can obtain through this indirect alignment. If this bound is less than the score of the best alignment we observed so far, we discard that indirect alignment. Otherwise, we improve indirect alignment to minimize indels and maximize the alignment score. Algorithm 2 presents a pseudo-code of our method. In the following, we elaborate on our method.



**Fig. 2:**
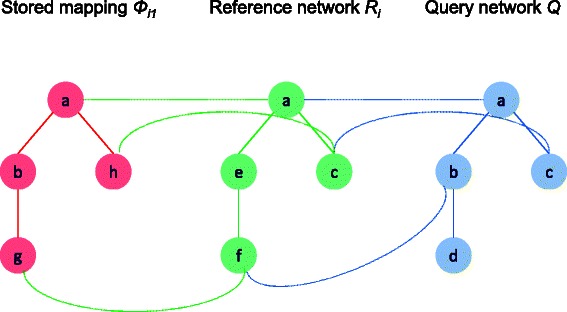
Overview of indirect alignment. At first, the query and reference networks *Q* and *R*_*i*_ are aligned. Let the matching function be *ψ*_*i*_. For instance, query node *b* is matched with the reference node *f* (i.e., *ψ*_*i*_(*b*)=*f*). In the reference generation phase, we stored the non-overlapping, significant mappings of *R*_*i*_ in the target network *T*. Let *ϕ*_*i*1_ be one of these stored mapping, and reference node *f* is matched with the target node *g* (i.e., *ϕ*_*i*1_(*f*)=*g*). Therefore, the indirect mapping of query node *b* is target node *g* (i.e., *ϕ*_*i*1_(*ψ*_*i*_(*b*))=*g*)

Given a query network *Q*, we first align it to each reference network $R_{i} \in \mathcal {F}$ optimally with 99 % confidence using the ColT algorithm (Algorithm 2, line 3). As both *Q* and *R*_*i*_ are small networks, this alignment has negligible computational cost. Let us denote this mapping by *ψ*_*i*_():*Q*→*R*_*i*_. For instance, *ψ*_*i*_(*u*)=*w* means that node *u* of *Q* is matched with node *w* of *R*_*i*_. If the alignment score between *Q* and *R*_*i*_ is below a given threshold, we conclude that *R*_*i*_ does not represent *Q* well. In this case, we ignore *R*_*i*_ and proceed to the next reference *R*_*i*+1_ in $\mathcal {F}$. If the alignment score is at least the given threshold, we study the resulting mapping from the nodes of *Q* to the nodes of *R*_*i*_ (Algorithm 2, line 4). Having computed *ψ*_*i*_, we iterate over each of the previously stored mapping in $\phantom {\dot {i}\!}\Phi _{i} = \{\phi _{i1}, \phi _{i2}, \ldots, \phi _{i\pi _{i}}\}$ to construct mappings from *Q* to *T* indirectly as follows (Algorithm 2, lines 5-12): Consider the *j*th significant, non-overlapping mapping function of *R*_*i*_ in *T*, *ϕ*_*ij*_():*R*_*i*_→*T*. For instance, *ϕ*_*ij*_(*w*)=*v* means that node *w* of *R*_*i*_ is matched with node *v* of *T*. The composition of these two mapping functions defines an indirect mapping between the nodes of *Q* and *T*, where *ϕ*_*ij*_(*ψ*_*i*_(*u*)))=*v* means that node *u* of *Q* is matched with node *v* of *T* indirectly through node *w* of *R*_*i*_ (Fig. 2 gives an overview of the indirect alignment on a toy example). We denote the set of such query nodes by *S*={*u*|*u*∈*V*_*Q*_, and *ϕ*_*ij*_(*ψ*_*i*_(*u*)))=*v* such that *v*∈*V*_*T*_, and *s* *i* *m*(*u*,*v*)≥0}. Similarly, let us denote the set of nodes in *T* which are indirectly aligned with those in *Q* through mapping *ϕ*_*ij*_ with *S* ^′^ (i.e., *S* ^′^={*v*|∃*u*∈*V*_*Q*_ such that *ϕ*_*ij*_(*ψ*(*u*))=*v*}). Next, we compute an upper-bound to the alignment score *U* *B*(*Q*,*ϕ*_*ij*_) imposed by *ϕ*_*ij*_ as follows (Algorithm 2, line 6). In the following, we describe our upper bound calculation strategy in detail.

**Upper bound calculation** We compute an upper-bound to the alignment score where the alignment subnetwork respects the imposed indirect mapping *ϕ*_*ij*_(*ψ*_*i*_()). Recall that if a query node *u* is in set *S*, it must match with a target node *v* in *T* (i.e., *ϕ*_*ij*_(*ψ*_*i*_(*u*))=*v*). If all the query nodes have an indirect mapping (i.e., *S*=*V*_*Q*_), we compute its alignment score. Otherwise, there is at least one query node *u* for which indirect mapping is undefined (i.e., *u*∉*S*). This leaves us with the following options: (i) We consider the node *u* as a deleted node and incur a deletion penalty, or, (ii) we find a potential target node *v* such that *s* *i* *m*(*u*,*v*) is large. Note that we may need to insert a node in the alignment subnetwork in order to get to such a suitable target node *v*, which will incur an additional insertion penalty. Since our goal is to compute an upper bound to the alignment score, we drop the topology constraint to calculate *U* *B*(*Q*,*ϕ*_*ij*_) to avoid indel penalty altogether as follows.

We construct a weighted bipartite graph *G*=(*V*_*Q*_, *V*_*T*_,*E*). For each query node *u*∈*S*, we insert the edge (*u*,*v*) in *E*, where *ϕ*_*ij*_(*ψ*_*i*_(*u*))=*v*. For each query node *u*∉*S*, we insert an edge between *v* and all nodes in *V*_*T*_−*S* ^′^. We assign the weight of all the edges (*u*,*v*) in *E* as *s* *i* *m*(*u*,*v*). We then use a maximum weighted bipartite matching algorithm to get the highest alignment score. We drop the topological constraint to get the upper-bound score *U* *B*(*Q*,*ϕ*_*ij*_) as high as possible when the alignment is limited to the indirect alignment *ϕ*_*ij*_(*ψ*_*i*_()). Since the upper-bound formula ignores the penalty incurred due to node insertions and deletions, it is guaranteed to be a true upper-bound for those set of nodes. Note however that there may be another subset of nodes in the target network which produces a higher alignment score than this upper-bound. To ensure that such alignments are not missed, we have more than one references to index different subsets of the target network.

Note that the gap between the upper-bound and the actual alignment score when the alignment is constrained to the set of nodes indexed by the corresponding reference can vary widely. The worst case scenario for our upper-bound happens when all the edges are missing between the nodes pairs in the target when they exist in the query. For instance, consider two query nodes *u*_1_ and *u*_2_ which have an edge between them. Also consider the two target nodes *v*_1_ and *v*_2_ which are aligned with *u*_1_ and *u*_2_ respectively by the upper-bound algorithm but there is no edge between *v*_1_ and *v*_2_. This will introduce node insertion penalty for all the nodes on the shortest path from *v*_1_ to *v*_2_. Thus the gap between the upper bound and the true alignment score depends on the target and query network topologies. That said, the value of the upper-bound score does not affect the correctness of our method. If the upper-bound is smaller than the current best alignment score, we skip this particular alignment since we already generated a better alignment using another indirect mapping. Otherwise, it can only introduce false positives at this stage. Our algorithm filters these false positives at the end of the query processing step when we generate an alignment subnetwork induced by the indirect alignment (Algorithm 2, lines 7 and 8).

**Induced alignment** If the upper bound is greater than the current best alignment score, we find an alignment subnetwork $T^{\prime } = (V_{T^{\prime }}, E_{T^{\prime }})$ that conforms to the indirect mapping. We call this *induced* alignment, since this alignment is induced by the indirect mapping found in the previous stage. Recall that in the dynamic programming solution, we compute the score by invoking each of match, insert and delete operation, and then choose the operation that produces the highest score. The indirect mapping governs which query and target network nodes these operations are applied to. Following describes how we extend the dynamic programming to take indirect mapping into consideration. Any query node *u*∈*S* aligns with node *v* of *T* if *ϕ*_*ij*_(*ψ*_*i*_(*u*)))=*v*. For any other node *u* ^′^∈*V*_*Q*_, we have two options, among which we choose the one with the highest score: (i) Do not match *u* ^′^ with any node (i.e., deletion of node *u* ^′^), (ii) Match *u* ^′^ with an unmatched node in *T*. In a similar fashion, any target node *v*∈*S* ^′^ only matches with the corresponding query node *u*∈*S* such that *ϕ*_*ij*_(*ψ*_*i*_(*u*)))=*v*. For any other node *u* ^′^∈*V*_*Q*_, we skip the match operation between *u* ^′^ and *v*. Similarly, we skip the insertion operation for any target node *v*∈*S* ^′^ to respect the indirect mapping. We further extend similar filtering conditions for other query nodes as well. Assume that (*u*,*w*)∈*E*_*Q*_ where *u*∈*S* and *w*∉*S*. The indirect mapping *ϕ*_*ij*_(*ψ*_*i*_(*u*))=*v* localizes the mapping of *w* with an adjacent node of *v* in *T*; or incurs an insertion. These extra conditions successfully skip many future unsuccessful invocations of dynamic programming and cuts down the running time significantly.

Indirect alignment also affects the number of colors needed in the color-coding algorithm. Since the query nodes in *S* are already mapped, we ignore the color of the corresponding target nodes. Thus, it suffices to use only as many colors as the unmapped query nodes instead of the total number of query nodes. This reduces the number of iterations needed to reach a certain confidence level exponentially with the number unused colors. Thus induced alignment improves the overall running time of the query processing method by avoiding many unsuccessful dynamic programming invocations and reducing the number of iterations for a certain confidence value.

## Results and discussion

In this section, we evaluate the performance of our method through extensive experiments. We compare our method to ColT [10] since ColT has the best running time performance among existing methods which provide provable confidence level in the optimality of the result. We set the confidence level of ColT to 99 % in our experiments. We measure the *running time* and the *alignment score* of the alignment subnetworks. We describe the experimental setup in detail below.

**Target network** We use real protein-protein interaction networks (PPI) as target networks in our experiments. More specifically, we use the PPI networks of *Escherichia coli*, *Helicobacter pylori*, and *Mus musculus* from the MINT [37] database. Among them, the mouse (*Mus musculus*) PPI network is the largest with 1,346 proteins and 1,659 interactions. *E. Coli* network has the smallest number of proteins, but it has the highest interactions density with 4.7 interactions per protein (i.e., $\frac {2 \times 1460}{701}$) on the average among the three networks.

Additionally, we use the PPI networks of *Drosophila melanogaster* (fruit fly) and *Saccharomyces cerevisiae* (yeast) for cross-species network query analysis. We use the networks downloaded from DIP database [38]. We further enriched the fly network by adding interactions obtained from Stanyon *et al.* [39] and FlyGRID [25]. The fly PPI network contains 7,481 proteins and 26,201 interactions among them. The yeast PPI network contains 4,738 proteins and 15,147 interactions among them. Table 2 summarizes the number of nodes and interactions of these networks.

**Table 2:** Summary of the target networks

Organism	Number of nodes	Number of interactions
*Escherichia coli*	701	1,640
*Helicobacter pylori*	733	1,507
*Mus musculus*	1,346	1,659
*Drosophila melanogaster*	7,481	26,201
*Saccharomyces cerevisiae*	4,738	15, 147

**Query network** We create each query network by performing random walk over the given target network. More specifically, we first create a query set of 10 networks for each target network where each query network has six nodes (rationale for the size is discussed in Section ‘Index creation’). We also create additional query sets from each of these query sets by randomly perturbing the topological and homological characteristics of the query in these sets. We describe the network perturbation model we use below.

(i) (Topology) In order to alter the topology of a query network, we either insert a new node into or remove an existing node from that query network. In order to insert a new node *w*, we either insert it as a leaf node with the incident edge, or randomly pick an edge (*u*, *v*) from the query network. In the former case, we randomly choose a node *v* from *Q* and then insert the new node *w* by adding edge (*v*,*w*). In the later case, we replace the edge (*u*, *v*) with the edges (*u*, *w*) and (*w*, *v*). Deleting a node from the query is tricky since we must ensure that the network remains connected after removal. To delete an existing node *w*, we check the degree of *w*. If its degree is 1 (i.e., a leaf node), we simply remove *w* along with the edge incident to it. If its degree is two, then we remove *w* along with its incident edges and insert a new edge (*u*,*v*) between the pair of nodes *u* and *v* that were connected to *w*. Thus, by inserting and removing a node from the original query network, we create 10 additional query networks of seven nodes and 10 networks of five nodes for each target network.
(ii) (Homology) In order to alter the homological similarity of the query network and the target networks, we introduce a given amount of noise to the amino acid sequences of the query nodes. More specifically, given a mutation percentage *μ*%, we iterate over each amino acid of each protein in the given query network and replace the amino acid with a different one with *μ*% probability. For each of the query sets described above, we create homologically mutant query networks for *μ*= 0 % (i.e., no sequence mutation), 5 %, 10 % and 20 %. This way, for each query network, we introduce four different query networks with the same topology, but different amino-acid sequences for the constituent proteins.

In summary, we create a total of three topological and four homological variants of queries, leading to 3×4=12 query sets for each target network. With 10 queries in each query set for each of the three datasets, we experiment with totally 360 (i.e., 3×12×10) query networks covering a broad spectrum of topological and homological characteristics.

For cross-species network alignment query, we create query networks from the mitogen-activated protein kinase (MAPK) pathways of human, fly and yeast from the KEGG database [40].

**Index creation** Recall from Section ‘Index creation’ that in order to ensure that aligning a query and a reference network is computationally cheap, reference networks should be small and have similar (not necessary identical) size as the query networks. Following from this observation and the limits in the query size, we generate reference networks of six nodes (i.e., the same size as our original query networks). For each target network, we create three reference sets with coverage values equal to 60 %, 70 %, and 80 %. The initial reference set generation time with 80 % coverage for *H. pylori, E. coli* and *M. musculus* are 2.6, 4.6, 5.4 hours, respectively. The running time to generate the final reference set from the initial reference set is less than a second for each organism.

**Statistical significance of an alignment subnetwork** We measure the significance of the score of an alignment in terms of its *z-score*. We compute the z-score of an alignment between two networks *Q* and *T* as follows: let *s* be the score of their alignment. We create a large number of random alignments between *Q* and *T* by randomly mapping the topology of *Q* over *T*. The scores of these random alignments denote the *null distribution* of possible mappings of *Q* and *T* while preserving alignment topology. Let us denote the mean and the standard deviation of these alignments with *μ* and *σ* respectively. We compute the z-score of the alignment using as $\frac {s - \mu }{\sigma }$. In our experiments, we construct 10,000 random alignments for this purpose for each alignment.

**Implementation details** For each protein in the target networks, we download the corresponding amino acid sequence from the Uniprot [41] database. We use BLAST+ to compute the homological similarity between pairs of nodes in the query and target networks using their corresponding protein sequences. We set the similarity score between nodes as the normalized negative logarithm of E-value returned by blastp. Note that, one can replace this function with any other similarity function such as the coherence of the GO terms between pairs of nodes. We implement our method in C++. We fix the possible number of insertion *n*_*ins*_ and deletion *n*_*del*_ to at most two as per Dost *et al.* [6]. We implement maximum weight bipartite matching using the Hungarian method [42].

**Environment** We performed all experiments on a Linux server that has 3 GB RAM and AMD Opteron dual core processors running at 2.2 GHz.

### Effect of index selection strategy

Recall that we create our index structure in two steps where the first step aims to maximize network coverage while the latter aims to minimize the index size. In our first experiment, we evaluate the contribution of each of these two steps in the performance of our method.

**Fig. 3:**
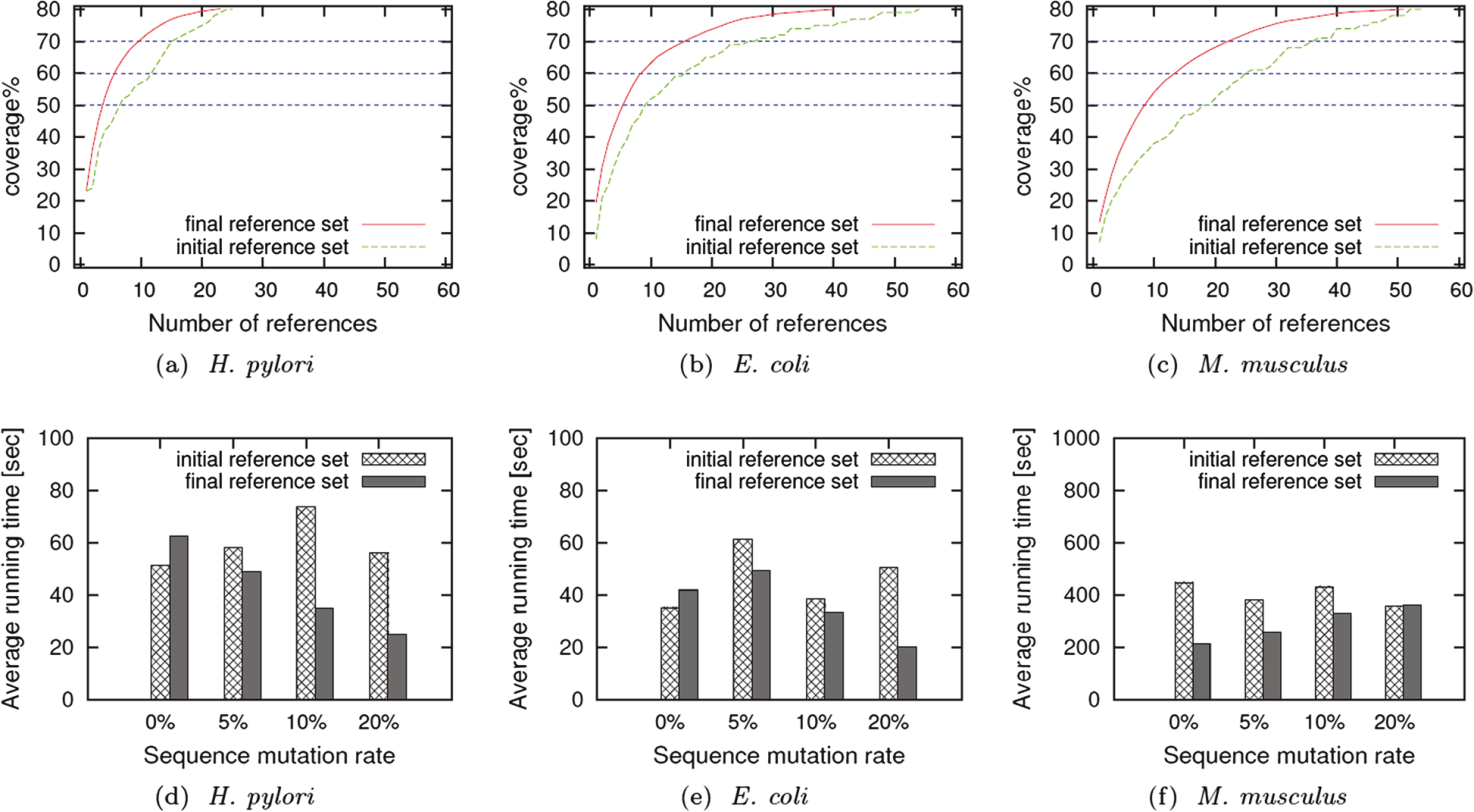
Figures (**a**), (**b**), and (**c**) show the coverage values obtained for growing number of reference networks on *H. pylori*, *E. Coli*, and *M. musculus* datasets respectively for initial and final reference sets. Figures (**d**), (**e**) and (**f**) report the average running time needed to query each of these three networks using the initial and final reference sets

We first report how the coverages of the initial (Step I) and final (Step 2) reference sets grow with increasing reference set size. Figure 3(a), (b) and (c) show the results for the three target network datasets. First of all, we observe that our index structure obtains very high coverage values (up to 80 %) by using a very small number of references (30 to 60). Our results also demonstrate that the final reference set requires much fewer reference networks than the initial set to reach the same coverage of the target network (coverage = 50 %, 60 %, and 70 % are highlighted). For example, in *E. Coli*, the final reference set needs only 16 references to reach 70 % coverage, whereas the initial set requires 26 references to reach the same coverage. The gap between the two sets reduces as the coverage of the final set grows beyond 80 %. However, we were able to generate the final reference set of *E. Coli* with 80 % coverage using only 75 % references from the initial set.

Next, we measure the average query processing time for different query sets and target networks using the initial and final reference sets. Here, we report the results using the original query networks (no insertion/deletion) and vary the sequence mutation rate to 0 %, 5 %, 10 %, and 20 %. We perform the experiments using index structures with different coverage values. In Fig. 3(d), (e) and (f), we report the results by setting the coverage to 60 %. Results for other coverage values have similar trends (figures not shown). For each of the three target organisms, we observe that the final reference set yields much faster query processing time in almost all query sets. For example, with 20 % sequence mutation on the query set, final reference set processes the query 2.5 times faster than initial reference set in *E. coli*. In some cases, initial reference set performs better than the final reference set, possibly due to finding a good alignment subnetwork in the early phase of the processing. The gain in running time in those cases is insignificant. These set of experiments suggest the potency of the final reference set over the initial reference set. Following from these observations, in the rest of our experiments, we only report the results using the final reference set.

### Comparison with the state-of-the-art method

Aligning a query with a target network is a computationally expensive task. Our indexing technique aims to reduce this cost. In this section, we evaluate our method’s success and limitations towards that goal. To do that, we compare the performance of our method to that of ColT in terms of running time and z-score of the alignment score. We use the following metric to compare the running time performance between the proposed method and ColT: $\text {speed-up} = \frac {\text {running time of ColT}}{\text {running time of the proposed method}}$. Larger speed-up values indicate better performance of the proposed method.

**Fig. 4:**
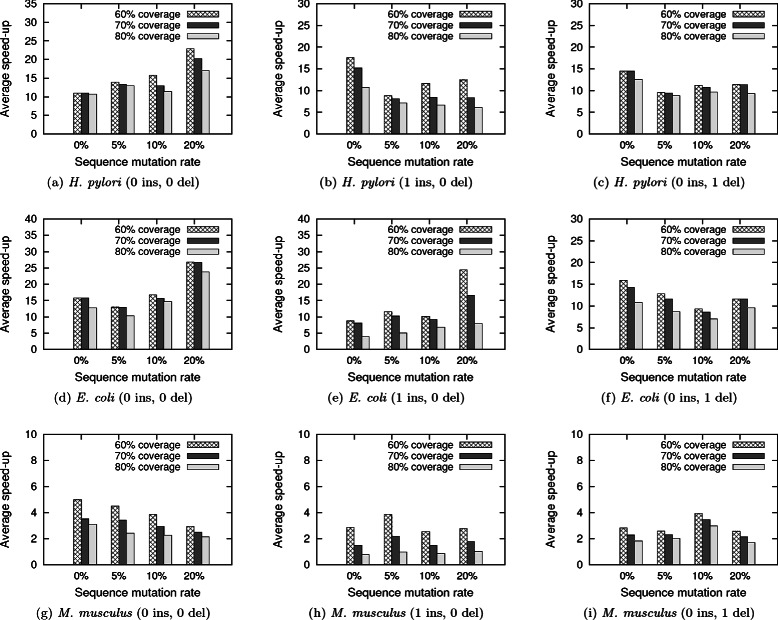
The average speed-up of the proposed method over an existing method, ColT. Results are reported by varying the query set through both topological and homological perturbations. Figures (**a**)-(**c**) show results for *H. pylori* network. Figures (**d**)-(**f**) show results for *E. coli* network. Figures (**g**)-(**i**) show results for *M. musculus* network

Figure 4 shows the average speed-up in running time obtained by our method for different query and index structure parameter settings. We observe significant speed-up in running time consistently for all target network, query set combinations. It also shows a general trend: the speed-up increases (i.e., the query processing time decreases), as the coverage of the index decreases. This is expected as the number of reference networks (along with its non-overlapping, significant alignment subnetworks) in the index increases with the expanding coverage values. This results in additional indirect alignments between the query and the reference networks, and invocation of expensive dynamic programming method. For instance, if we set the homology mutation *μ* to 20 % and use the query networks with seven nodes, we observe that our method with 60 % coverage runs 25 times faster than ColT in *E. coli* network. If we increase the coverage to 80 %, it runs eight times faster. In some cases, our method obtains high speed-up values independent of the coverage size. For instance, if we set the homology mutation *μ* to 20 % and use the query networks with seven nodes, we observe that our method runs 27 and 24 times faster with coverage set to 60 % and 80 % respectively in *H. pylori* network.

The speed-up in query processing time is desirable only if the resulting alignment score is significant. In our next experiment, we evaluate whether our method can find significant alignments despite filtering a massive portions of the target network. Table 3 presents the average z-scores of the alignments found by our method and ColT for each dataset and parameter settings. We observe that the obtained z-scores are very high in all settings. Even in the worst case our z-score is above 8, and thus, it is statistically very significant. Furthermore, the z-score of the alignment obtained by our indexing strategy is very close to the z-score obtained by ColT in many cases. For instance, z-score obtained by ColT and the index with 80 % coverage is almost always the same for each of the target network. In other cases, the obtained z-score of the alignment subnetwork is so high that it makes little or no difference to the quality of the alignment found by ColT. We see that the z-score increases as the coverage of the index increases. This is expected since additional coverage gives chance to explore further in the target network. These results suggest that our index is a preferable alternative to the existing methods, including ColT method as it finds statistically significant alignments with spectacular speed-up.

**Table 3:** Average *z-score* computed from the alignment score using different index coverage. We use $cover(\mathcal {F}) =$ 60 %, 70 % and 80 %. In all these cases, the alignment subnetworks are found to be statistically very significant

	0 insert, 0 delete				1 insert, 0 delete				0 insert, 1 delete				
							Index Coverage						
	Sequence	60 %	70 %	80 %	ColT	60 %	70 %	80 %	ColT	60 %	70 %	80 %	ColT
	mutation												
*H. Pylori*	0 %	28.99	28.99	28.99	28.99	24.92	25.18	27.49	28.32	26.16	26.16	26.80	26.80
	5 %	28.77	29.05	29.56	29.56	25.57	25.59	26.20	28.62	24.89	24.89	26.62	26.62
	10 %	25.49	28.82	30.15	30.15	22.53	26.43	27.90	29.22	25.26	25.73	27.42	27.42
	20 %	25.14	28.24	29.78	29.78	22.41	26.43	28.43	30.61	26.81	26.81	28.03	28.04
*E. coli*	0 %	30.25	30.25	31.13	31.13	24.01	24.11	28.93	29.60	25.38	25.38	26.64	26.64
	5 %	28.99	28.99	31.06	31.06	26.39	26.75	31.14	31.02	25.60	25.60	27.48	27.48
	10 %	26.53	28.62	30.10	30.10	21.68	24.99	25.42	30.76	23.84	26.26	27.79	27.79
	20 %	31.65	31.65	32.08	32.08	30.89	30.91	31.82	32.65	28.07	28.07	29.00	29.55
*M. musculus*	0 %	15.37	15.37	18.05	18.08	8.64	10.15	13.00	14.80	13.77	14.57	16.72	16.72
	5 %	12.46	12.61	17.49	18.34	8.86	9.97	10.40	15.28	14.93	15.42	16.93	16.93
	10 %	13.86	15.25	16.30	19.11	10.22	11.86	12.87	15.42	15.34	15.34	15.89	17.60
	20 %	14.04	15.51	20.45	20.47	9.11	11.10	11.40	16.66	14.99	15.67	18.90	18.90

### Effect of method parameters

In this section, we discuss the effect of the method parameters on the performance of the proposed method. We also evaluate the performance of the parameter values that affect the index and query sets. We vary the coverage of the index and measure the resulting running time and z-score of the alignment using all the query sets. We explain the effect of these parameters in detail below.

**Effect of coverage** Figure 4 shows the effect of coverage on the query processing time. Coverage of the index has a direct impact on the running time of our method. We observe significant speed-up in running time by using the index structure with different coverage values. More specifically, as we lower the coverage value slightly, we observe significant speed-up in query processing time using the index. This suggests that using a lower coverage value (around 60-70 %) leads to better running time performance. Next, we evaluate the effect of coverage on the z-score of the alignment. Table 3 shows that we find alignments with significant z-score using different coverage values. We also observe that the z-score of the alignment increases as the coverage increases. This is expected as increased coverage allows to finding alternate alignments at different parts of the target network. Therefore, using a higher coverage value is a good choice for more accurate results. We can find an easy balance between the two seemingly contradictory trends using this fact: the obtained z-score of the alignment remains very significant even with decrease in the coverage. For instance, for all possible parameter values in *E. coli*, z-score ranges from 20 and reaches above 30. We can choose a suitable value for coverage as per the need of the user.

**Fig. 5:**
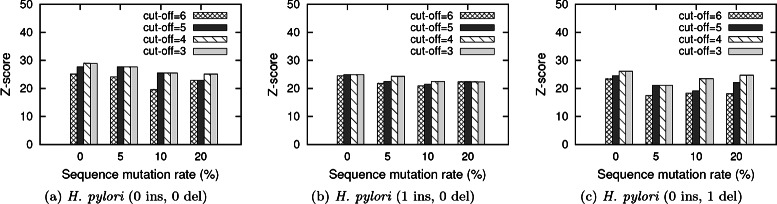
Average z-score of the alignments found by our method for query networks with varying homological perturbation parameters with different significance cut-off in the *H. pylori* network. Figures (**a**), (**b**) and (**c**) show the results with no indel, one insertion and one deletion of node respectively

**Effect of significance cut-off** In this experiment, we vary the significance cut-off values for index creation and observe its effect on the quality of the result. Figure 5 presents the effect of different significant cut-off values (varies from six to three) on the z-score for *H. pylori*. We observe similar results for the other two organisms as well (results now shown). In all these experiments, we obtain results with very high z-score values. We also observe that the z-score increases monotonically, as we decrease the significance cut-off values from six to three. Recall that the significance cut-off is used to store significant mappings of the reference networks. For smaller values of cut-off, we store more mappings of the reference networks in the index. As we process the given query network using these stored mappings, chances of finding the alignment subnetwork with high z-score value increases. Similarly, with larger significance cut-off values, we store fewer mappings in the index. As a result, it expedites the query processing time.

**Fig. 6:**
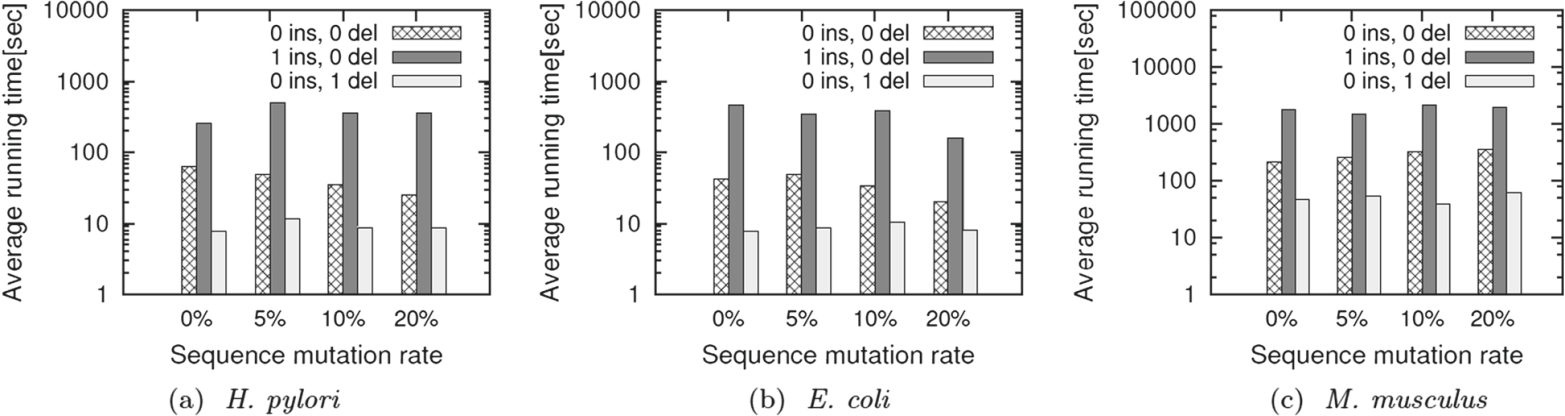
Average running time of our method to align the query networks with varying topological and homological perturbation parameters for the three target networks. Figures (**a**), (**b**) and (**c**) show the results for *H. pylori*, *E. coli* and *M. musculus* target networks respectively

**Effect of query parameters** We measure the effect of query size and homological deviation of the query network from the target network on the performance of our method. To measure the effect of query size, we use all the query sets (both original and perturbed) for this experiment. Figure 6 reports the running time versus the size of the query network when the coverage value is set to 60 %. We observe similar results for other coverage values as well. We observe that the running time increases as the number of nodes in the query increases. This is expected as the underlying dynamic programming is exponential on the number of nodes in the query network. So, it takes more time to compute the local alignments for larger query networks, as we iterate over the list of non-overlapping, significant mappings for a successful induced alignment (Algorithm 2, line 8). We also vary the amount of homological perturbation rate to 5 %, 10 % and 20 % in the query set. As we can see from Fig. 6, the running time does not vary much based on the mutation in the sequence of proteins. Since the mutation in the amino acid sequence is random, it changes the similarity score between the pair of query and target proteins arbitrarily. In short, topological mutation has more dramatic effect on the running time than homological mutation. Table 3 shows that the z-score of the alignment is very high, and independent of the change in method or query parameters (i.e., coverage, topological and homological mutation). This is a very desirable characteristic of our method. It shows that we can use our indexing structure in many different parameter settings and it still produces significant alignments.

### Cross-species alignment

So far, we extracted the query networks from an organism and employed our indexing method to find the alignment subnetworks in that same organism. Our experiments have demonstrated that our indexing method finds statistically significant alignment subnetworks in much faster running time. Now, we evaluate how well our indexing structure works when we perform cross-species alignment. In this experiment, we query a known pathway from one organism, and check if the alignment subnetwork in another organism (found by our indexing method) is meaningful. For this purpose, we extract query subnetworks from MAPK (mitogen-activated protein kinase) pathways in human, fly and yeast networks. We employ our method to find the alignment subnetworks in fly and yeast target networks and measure the statistical and biological significance of the results. We use the DAVID [43, 44] tool for functional annotation purpose.

We start by creating three small query networks of six nodes. We extract a small query network from classical human MAPK pathway that is responsible for cell proliferation and differentiation. We also extract a query network from yeast MAPK pathway that is involved in nitrogen starvation inducing filamentation. The third one is from fly MAPK pathway and is responsible for R7 photoreceptor cells in fly eye development.

**Fig. 7:**
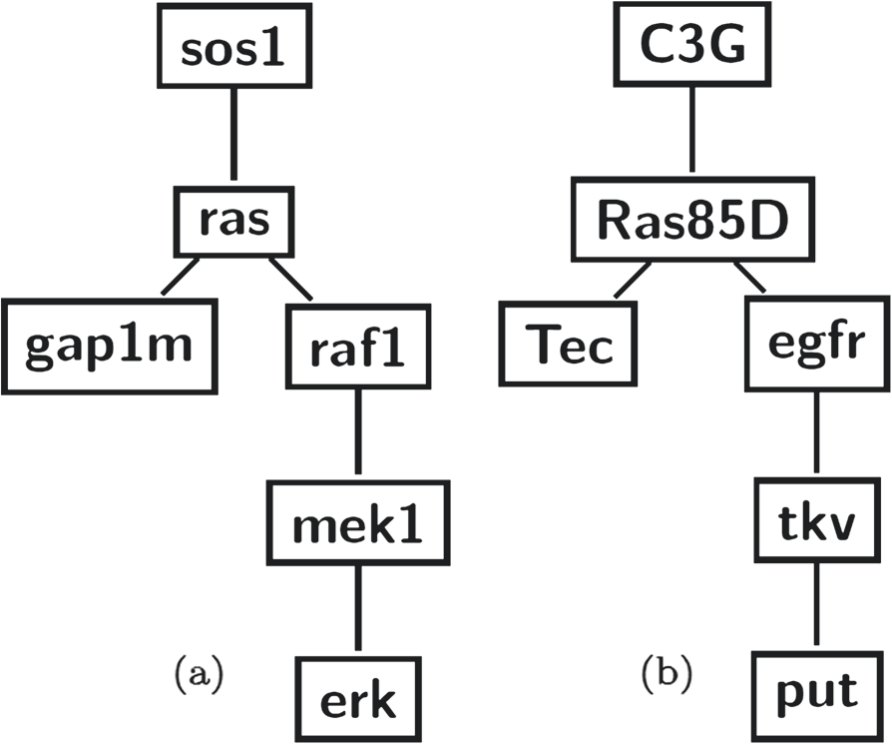
Cross species network query using index. Figure (**a**) shows a query network extracted from human MAPK pathway and (**b**) shows the corresponding alignment subnetwork in fly

We first align human MAPK query network against the fly PPI network using our indexing method. Figure 7 shows the query and the alignment subnetwork. The proteins Ras85D and Egfr in the alignment subnetwork are responsible for MAPK functions in particular. We use the DAVID tool to generate the functional annotation chart and in the ‘functional categories’, we find the term *kinase* as the most enriched one with *p-value* =4.2*e* ^−5^ which is significant. In the ‘Gene Ontology’ category, we find the term GO:0004672 (protein kinase activity) as the most significant one with *p-value* = 4.28*e* ^−4^. We also query the InterPro database [45] to functionally analyze these proteins by classifying them into families and predicting domains. We find that the term IPR000719 (Protein kinase, core) is the most enriched one with the *p-value* =9.09*e* ^−5^. Additionally, we also measure the statistical significance of the alignment subnetwork in terms of z-score. The z-score value of 17.94 also shows that the mapping is very significant. This shows that the human query network used in this experiment maps to functionally conserved regions in the fly network. We then align the yeast query network against the fly network and use the DAVID tool to analyze the result. In the ‘Gene Ontology’ category, we find the term GO:0004672 (protein kinase activity) appearing with *p-value* =4.28*e* ^−4^. In the ‘functional categories’, we find the term *kinase* with *p-value* =2.6*e* ^−3^. Similarly, when we query the matching proteins in the InterPro database for predicting families and domains, we find the term IPR000719 (Protein kinase, core) as the most enriched one with *p-value* =9.09*e* ^−5^. The z-score of the alignment subnetwork is 15.0 which shows that the alignment subnetwork is also statistically meaningful. These results demonstrate that query networks extracted from human and yeast MAPK pathways match with functionally conserved regions in fly.

**Fig. 8:**
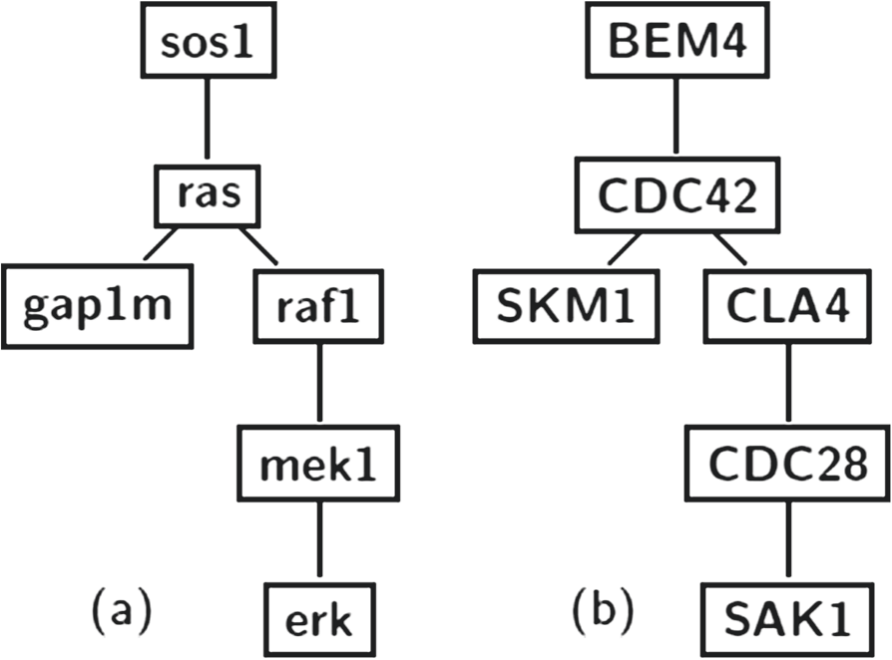
Cross species network query using index. Figure (**a**) shows a query network extracted from human MAPK pathway and (**b**) shows the corresponding alignment subnetwork in yeast

We then repeat the experiment with human and fly query networks against the yeast network. Figure 8 shows the human query and its alignment subnetwork in yeast. The protein CDC42 in the alignment subnetwork is responsible for MAPK function in particular. We use the DAVID tool to generate the functional annotation chart and in the ‘functional categories’, we find the terms *serine-threonine-protein kinase* and *kinase* as the most enriched ones with *p-value* =6.3*e* ^−5^ and 2.9*e* ^−4^ respectively. In the ‘Gene Ontology’ category, we find the terms GO:0004674 (protein serine/threonine kinase activity) and GO:0004672 (protein kinase activity) as the most significant ones with *p-value* =2.73*e* ^−4^ and 3.12*e* ^−4^ respectively. We also query the InterPro database [45] to functionally analyze the proteins and find all the top terms are kinase related (IPR002290, IPR017442, IPR008271, IPR000719, IPR015750, IPR017441 etc.), where IPR000719 (Protein kinase, core) appears with the *p-value* =6.00*e* ^−5^. The z-score of the alignment subnetwork is also 12.02. This shows that the human query network used in this experiment maps to functionally conserved regions in the yeast network. We then align the fly query network with the yeast PPI network. When we query the InterPro database to functionally analyze the proteins, we find the terms IPR017442, IPR017441, IPR008271, IPR000719 as the most enriched ones (all are kinase related). The term IPR000719 (Protein kinase, core) appears with the *p-value* =8.00*e* ^−3^. Our method finds statistically significant alignment subnetwork (i.e., z-score = 12.21). *These experiments show that our indexing method finds alignment subnetworks that are statistically significant and functionally coherent.*

## Conclusion

In this article, we proposed an indexing structure that accelerates network queries on large target networks. We developed a reference based indexing on the target network. Our index consists of small networks named ‘references’ carefully chosen from the target, along with their significant, non-overlapping mappings to the target network. Given a query network, we first assess each reference network for its similarity with the query network. If it has the potential to produce the alignment subnetwork, we generate an indirect alignment from the query to the target using the reference, and process its stored mappings to find the alignment subnetwork. Our experiments suggest that the running time of our method is dramatically better than the existing approximate methods. We also observed that the alignment subnetworks found by our method are statistically and functionally significant.
